# Professional socialisation of nursing students in a collectivist culture: a qualitative study

**DOI:** 10.1186/s12909-019-1690-z

**Published:** 2019-07-09

**Authors:** Jung Jae Lee, Sook Ching Yang

**Affiliations:** 1 School of Nursing, The University of Hong Kong, 4/F William M.W. Mong Block, 21 Sassoon Road, Pokfulam, Hong Kong; 2 Vascular Surgery, The Royal Infirmary of Edinburgh, 51 Little France Crescent, Old Dalkeith Road, Edinburgh, EH16 4SA, UK

**Keywords:** Nursing, Education, Healthcare, Clinical, Placement, Professional, Socialisation, Culture, Collectivism, Curriculum

## Abstract

**Background:**

Beyond the formal curriculum of skill attainment, nursing students are able to undergo the professional socialisation process in clinical contexts and establish their identity as healthcare providers. However, the cultural context that affects the socialisation process in clinical placements is less discussed. We aimed to explore nursing students’ learning and professional socialisation during clinical placements by considering the socio-cultural contexts in South Korea.

**Methods:**

A grounded theory approach was used for this research. Four rounds of in-depth and intensive interviews were carried out, with the recruitment of 16 nursing students, four nurses and two university lecturers in South Korea (29 interviews in total). A constructivist grounded theory framework was adopted to analyse the interview data. NVivo 11 was used to manage the interview data for analysis.

**Results:**

The researchers identified the process of learning and professional socialisation under three core themes: 1) Struggling at the bottom of the hierarchy, 2) Acceptance and conformity, and 3) The need for ‘nunchi’ (in Korean, it means to study the atmosphere and discover the embedded intention of others’ behaviour). The results offered insights into the challenges encountered by nursing students on clinical placements and how students attempt to adapt and conform to the difficulties encountered in clinical education to maximise their learning and for their professional socialisation. The significance of the hidden curriculum was discussed.

**Conclusions:**

While experiential learning is a great opportunity for students to build on their coping skills and professional socialisation, a lack of support can result in failure to manage the hidden curriculum and theoretical and practical skills. Nursing educators therefore need to orientate students to the professional culture prior to beginning clinical placements.

**Electronic supplementary material:**

The online version of this article (10.1186/s12909-019-1690-z) contains supplementary material, which is available to authorized users.

## Background

Clinical education is an essential component of healthcare training, including the nursing field [1–3], whereby students apply their theoretical knowledge and build their practical skills to achieve competency for practice. However, beyond achieving the aims of the formal curriculum, there is a growing emphasis on the need to prepare students for working in real clinical contexts [4]. John Dewey [5], known for the concept of ‘learning by doing’, contends that learners’ experiences of genuine contexts and their awareness of those contexts are invaluable parts of experiential learning. Through such experiences, students are able to learn not only the practical skills of the profession but its roles, values, and norms to develop their professional identity as healthcare providers [2, 6–8].

However, earlier studies have found that students in healthcare fields encounter difficulties in achieving their learning goals during clinical placements. These difficulties include: negative interpersonal relationships with healthcare professionals [9–11]; feelings of powerlessness in clinical environments [9, 11, 12]; exclusion from professional groups [9, 10]; insufficient time provided for clinical education [11, 13, 14]; the negative impact of professionals’ heavy workload on clinical education [11, 15] and emotional distress caused by any or all of these concerns [9–11, 16]. Disrupted professional socialisation processes caused by any one or combination of these difficulties can result in feelings of a lack of belongingness to the students’ professional group [10].

South Korea is regarded as a collectivism-dominant country whereby Confucian ideas and practices are integral to its society and culture [17]. Group membership and group harmony is particularly emphasised in South Korea, therefore a lack of belongingness can eventually lead to poorer motivation to continue in the profession [10]. Moreover, as group welfare is prized over individualism, hierarchy and discipline is likely to be more highly valued in South Korea, as opposed to Western societies [18]. However, it was found that discipline could also significantly hinder clinical education and delay students’ engagement in the clinical placement [19, 20]. While there are many studies exploring the concept of professional socialisation, there are few that factor in the effect of cultural context on nursing students’ professional socialisation during clinical placements, particularly in collectivist societies like South Korea.

Thus, the aim of this study is to explore nursing students’ experiences of their learning and professional socialisation during clinical placements through consideration of the socio-cultural contexts in South Korea.

## Methods

### Design

Constructivist grounded theory (CGT) was used to inform this qualitative study as it allowed identification of participants’ individual processes and their interactions with social contexts by co-construction of knowledge between the researchers and the participants [21, 22].

### Study setting

This study was conducted by recruiting nursing students in Seoul, Korea. Universities in Korea run four-year undergraduate nursing programmes and the students usually start their clinical placements in their third year. Nursing students in Korea need to complete a minimum of 1000 h of clinical placement experience to become a qualified nurse [23].

### Participants

Purposive sampling was initially used to recruit nursing students who were in their final year of their undergraduate nursing baccalaureate programme as they would have had at least one experience of clinical placement. We e-mailed four nursing schools and asked for support in the recruitment for this study. They shared information about the study with their students and introduced 15 nursing students who met the inclusion criteria and volunteered to participate this study. Ten of the 15 students were finally recruited for the initial interviews.

After commencing the initial round of data collection and analysis, further participant selection was achieved through theoretical sampling [21]. Theoretical sampling involves selection of participants who are best positioned to provide data that will allow further examination and refining of developing categories until theoretical saturation is reached [21]. Through theoretical sampling, we recruited six more nursing students, four nurses and two university lecturers (who have had experience in teaching or supervising nursing students), to provide in-depth and holistic viewpoints of the interview topics. We did this by contacting two of the universities the initial participants were studying at and two lecturers and six more nursing students volunteered to participate after hearing about the study from the nursing Head of Department. We also emailed two large tertiary hospitals in Seoul and recruited four nurses who expressed interest in the research.

### Data collection

**Fig. 1:**
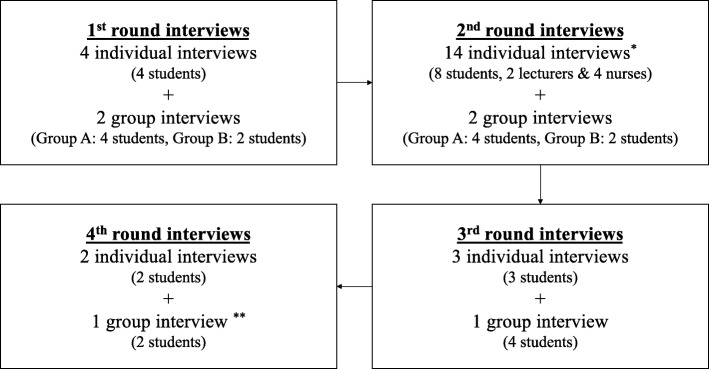
Interview rounds (^*^4 students, 2 lecturers & 4 nurses were newly recruited; ^**^ 2 students were newly recruited)

A total of 29 interviews (a mixture of 23 individual and six group interviews) were conducted in four rounds from April 2013 to June 2014 using theoretical sampling (see Fig. 1). In the first round, four students for individual interviews and six students for two group interviews were recruited. Additional to the 10 students from the first round, four students, two lecturers and four nurses were newly recruited for individual interviews in the second round (14 individual and 2 group interviews). Among the 14 total students from the second round, three students and four students were re-recruited for individual interviews and a group interview respectively for the third round (3 individual and 1 group interviews). Finally, for the fourth round, two students who participated in all the former rounds were re-recruited for individual interviews, with two newly-recruited students for a group interview (2 individual and 1 group interviews).

Interviews were semi-structured with open-ended questions (see the Additional file 1 for the interview guide) [21], and were all conducted by JJL in universities or hospitals that reflected educational contexts familiar to the participants. Intensive interviewing was employed due to its suitability to CGT methodology and in achieving the research aims by obtaining information of a certain topic, the interviewee’s experiences of the topic and his/her reflections on those experiences [21]. Interviews each lasted up to two hours and were recorded using a portable voice-recorder. Memos were taken during and after interviews to reflect the interviewer’s ideas and experience of the participants’ answers, allowing the preservation and development of research topics [21].

### Data analysis

Due to nature of CGT requiring consistency in the analysis process, the data analysis was initially conducted by JJL and then further discussed with SCY. The analysis process began immediately after each interview and the summary and interpretation of each interview transcript were sent to each participant to seek their confirmation. Taking to account participants’ opinions of the researcher’s interpretation of the data is in keeping with a key concept of CGT methodology, which is the co-construction of knowledge (or meaning) [21].

Interview recordings were transcribed and NVivo 11 was used to manage data and support analysis. After using initial coding to abbreviate data, focused coding was used to develop core categories. Finally, theoretical coding was used to specify possible relationships between developed core categories. Data was analysed using a CGT approach, with constant comparison analysis between data, codes, memos and categories [21]. The analysis process was continued until theoretical saturation is reached.

Meanwhile, language should be carefully considered in qualitative research to preserve underlying meanings [24]. Thus, the translation process of interview transcriptions in Korean to English was carefully designed. A translation team that comprised of five bilingual persons (i.e., in English and Korean) was formed for the translation and back-translation process.

### Reflexivity

As to reflexivity [21], JJL has an in-depth understanding of general and educational culture in Korea, having graduated from a nursing degree course and worked as a nurse in Seoul for five years. Moreover, JJL has also worked as a nursing educator and has significant experience in conducting qualitative research. Such experience influenced the interpretation of interview data collected here. Particularly, the memoing process played an important role in reflecting JJL’s experience and paradigm in the data interpretation.

### Rigour

Rigour in qualitative research is crucial in ensuring the value of the results [25]. This research was systematically conducted according to Charmaz’s [21] criteria, as shown in Table 1.

**Table 1:** Rigour of this research

Credibility	
- To increase the familiarity of the research topics and settings, the attempt was made to consider the contextual backgrounds in which the research was taking place. - Through theoretical sampling and memoing, the research achieved theoretical saturation with sufficient data. - Throughout the theoretical coding using constant comparisons, the research was able to identify the relationships between the categories that emerged. - A systematic process was adopted to analyse data as well as minimise translational limitations, to ensure credibility.	
Originality	
- In the light of CGT methodological techniques, this research was able to discover new categories and theoretical frameworks concerning nursing students’ learning absent from earlier work. - The originality of new findings was confirmed during literature reviews.	
Resonance	
- As theoretical saturation was achieved, the researchers believe the students’ experience of nursing education in Korea was sufficiently explained. - A series of four interview stages was conducted to define taken-for-granted meanings on the participants’ part. - The research was carried out with the full cooperation of participants, who examined the findings and then independently confirmed the findings (i.e., member-checking).	
Usefulness	
- The research findings were grounded in the data collected. - It included both the participants’ general and specific experiences of nursing education. Thus, it is expected that the findings are practical and can be applied to improve nursing education. - However, further studies will be required to confirm the findings because of the originality of the present research.	

## Results

### Demographic characteristics of the participants

The mean age of 16 nursing students was 21.25 years (SD = 1.81), while the mean age of four nurses were 37 years (SD = 8.91) in this study. The 87.5% of nursing students and all nurses were female. The mean working experience of the nurses in clinical environments was 12.25 years (SD = 8.50). In addition, a 33-year-old female lecturer and a 53-year-old male lecturer who had worked for three years and 19 years respectively in nursing schools were recruited.

This research uncovered the process of learning and professional socialisation nursing students undergo during clinical placements. The subthemes from this process are “Struggling at the bottom of the hierarchy”, “Accepting and conforming”, and “The need for nunchi”.

### Struggling at the bottom of the hierarchy

South Korea is a collectivist society based on Confucianism and has a culture of hierarchical organisation. Social positions such as age, job titles and work experiences within an organisation determine the interpersonal relationships, and that relationship was vertical.

*The relationship between juniors and seniors is very unilateral indeed. The seniors always have her/his own way in dealing with the juniors, but the juniors cannot express their bad mood to the seniors. This situation does not apply only to nurses, but rather, applies across the Korean society.* (Miran, Nursing student)

Nursing student participants learned about the importance of a social hierarchy within the clinical context, and particularly within nurse groups, through the strict discipline imposed on them and the consequences reaped if that discipline was breached.

*If I don’t maintain the discipline … I will definitely get scolded by the nurses* (Yoonjin, nursing student)

*Discipline within the nursing community is strict, like the military … [For example,] nursing students shouldn’t talk in hospital elevators, and shouldn’t eat while walking …* (Miran, nursing student)

The student participants established through the discipline imposed on them that they were in the lowest social position in the nurse group hierarchy. By being at the bottom of the hierarchy, their roles and expected behaviours as students were clearly distinguished from professional nurses.

*I am not the nurses’ colleague yet and I'm the low man on the totem pole. Due to [our] society’s traditional culture, the vertical ranking order and the relationship between the higher and lower-ranked, we have to be careful of our behaviours.* (Sarang, Nursing student)

Due to the position at the bottom of the hierarchy, the student participants struggled to learn during their clinical placements.

*I feel that we are the weak ones when trying to absorb nursing knowledge [in clinical environments]. Nurses communicate with us in a one-sided way [because we are the weak ones].* (Dahee, nursing student)

*Nursing students are at the bottom if nurses are at the top in the vertical relationship... I think we are [like] servants who learn once in a while [in clinical environments]?* (Yoonjin, nursing student)

However, it should be noted that while the vertical relationship and discipline in clinical contexts were not taught as part of the formal curriculum, the student participants were aware that they are generally expected to behave in a disciplined manner during clinical placements.

*There are invisible rules that nursing students should obey during clinical placements.* (Kuntaek, nursing student)

*Nobody tells us that we should maintain proper attitudes and participate in clinical placements sincerely, but we cannot act otherwise.* (Ari, nursing student)

Nurse participants confirmed this and explained that they themselves had gone through the same process as the student participants.

*We [ourselves] have learnt! Nursing students should always have proper attitudes and be enthusiastic … I think it is a custom [in nursing fields]. We have a stereotypical way of thinking, like ‘students shouldn't do that’.* (Hansol, nurse)

### Acceptance and conformity

Despite the student participants’ struggle to learn at the bottom of the hierarchy, they expressed acceptance of the situation.

*There are very few things we can do as students during clinical placements. I find this frustrating. However, I understand I can’t amend this situation as a student. ... It is a shame that I really want to learn more in the clinical environments, but I can’t.* (Dahee, nursing student)

Acceptance of the challenges to their learning during clinical placement was attributed to their cultural background.

*It is the universal human relations in Korea... It would be easier if the students could ask [the nurses their questions] with the mentality of “As a student, I will learn whatever there is to learn!” But, it is actually really difficult [for them to do so]... I think it would be nice for students if we could change this Korean culture bit by bit.* (Jongwon, University lecturer)

Their decision to conform to the hierarchy and the expectations placed on them could be seen through their awareness of the importance of clinical placements for their professional development and their desire to learn.

*I might not know what the nursing processes are, how to make nursing diagnoses, and so on, if I only learn theory without the clinical placements. Now, I know these because I have gained experience through the placements.* (Hyemin, nursing student)

*I think the clinical environments are ideal [for learning nursing practice] … I really hope I have more opportunities to learn in clinical environments.* (Kuntaek, Nursing student)

### The need for nunchi

In response to their clinical education situation, all student participants used the word ‘nunchi’. Nunchi is a Korean word and it literally means ‘eye measurement’ [18] in English. Nunchi can be ‘studied’ – which is the effort to understand others in a certain environment by studying the atmosphere and discovering the embedded intention of others’ behaviour – or ‘felt’, whereby the receiver believes that his or her counterpart is expressing discontent through indirect messages. This is an individual’s social interpretive process of others from a first-person perspective and a subjective communication method.

Student participants used nunchi to make an active analysis of nurses’ behaviours and the clinical atmosphere before deciding on their own behaviour and response in the hierarchical clinical context. This was a coping strategy to increase learning opportunities by influencing the level of rapport between student and nurse. Through the accurate deduction of the nurses’ nunchi and accordingly-adjusted behaviour, student-nurse rapport could improve, reducing the negative effects of nunchi and increasing the opportunities for learning.

*I should first study the nurses’ nunchi [before asking]. Because, I can learn something from the nurse when I correctly study her using my intuition …* (Dahee, nursing student)

*During my clinical placement, I followed and aided nurses by studying the nurses’ nunchi well. As a result, I became closer to the nurses. After becoming closer to the nurses, the wall between the nurses and myself was broken down little by little. So, I didn't need to study the nurses’ nunchi as much, I could make small jokes with them, and I could comfortably do and enjoy my placement. The nurses also taught us a lot. So, it was of much benefit to me.* (Yoonjin, nursing student)

Nurse and lecturer participants of this research also acknowledged and advocated nursing students’ use of nunchi during clinical placements. They believed that it was inherent to the hierarchical Korean culture and essential for nursing students in their preparation to become fully qualified nursing professionals in South Korea.

*The reason why students feel nunchi is because they would believe that the nurses’ social position is hierarchically higher than their own. It is the universal human relationship in Korea.* (Jongwon, university lecturer)

*Students should learn nursing culture and develop the ability to analyse the atmosphere [during clinical education] … I think it is the process of becoming a nursing professional. I am sure that this ability will be useful for them when they work in hospitals.* (Nayoon, nurse)

Studying nunchi was not part of the formal curriculum. Yet, like the nurse and lecturer participants, the student participants believed that studying nunchi was related to their future professional career. Although the key purpose of clinical education for nursing students in the Korean formal curriculum was to learn nursing practice within clinical contexts, the students interviewed for this research believed otherwise. They thought that adjusting to the clinical atmosphere and learning the social norms of the profession within their shared cultural context – their professional socialisation – was the main purpose of clinical placements. They believed that knowing how to use nunchi in the clinical context was crucial not only to their learning but also to their professional socialisation to become professional nurses, as it allowed them to communicate more effectively with nurses.

*Clinical placements are for me to familiarise myself with the clinical environment, and in preparation for working as a nurse, to gain indirect experiences [of nursing practice] and [experience] nurses’ hierarchical culture.* (Hyemin, nursing student)

*I have developed some nunchi, so I now know whether I can sit down, touch this, say this, and so on. Because of that, it is less tiring than before. In the past, it was a lot harder on me emotionally than physically. If the nurse scolds me just once, I would be distressed all day long. But now it is not like that, because I got used to the clinical environments and thus could enhance my nunchi skill.* (Dahee, Nursing student)

*As days went by, I learnt how to communicate [with nurses].* (Eunju, Nursing student)

## Discussion

This research explored the struggles that student nurses encounter with their learning and professional socialisation in a hierarchical clinical context. It was discovered that they come to accept the challenging circumstances of their learning situation in clinical contexts, conform to it, and used a specific strategy called nunchi to overcome those challenges. It is noted that learning about the hierarchy in nurse groups, their need to accept and conform to their circumstances, and using nunchi as a strategy to gain acceptance in nurse groups and receive teaching were not explicitly taught or discussed prior to or during clinical placements.

Although the student participants struggled with the discipline, they accepted their situation by acknowledging the influence of their cultural background. Agreeing to conform to the negative learning conditions can be interpreted as their resilience to continue their learning process. The resilience the student participants possessed can be defined as an internal process of using personal factors to protect themselves from perceived stressors and, in doing so, help them to cope and adapt to a variety of challenging situations [26]. In this research, the student participants demonstrated resilience in using nunchi to achieve stronger rapport with the nurses despite the difficulties they experienced in reaching the nurses’ expectations. Persisting with their efforts to receive teaching also assisted the process of inclusion in nurse groups [27].

Clinical placements provide students with opportunities to acquaint themselves with the real-life nursing profession [20, 28, 29]. However, the clinical context is initially unfamiliar and difficult for Korean students, as most enter university directly after secondary school. They therefore tend to be more cautious and passive in unfamiliar social environments, compared to the secure and supportive environments of school and homes. Moreover, some studies [30, 31] insisted that the students required acceptance from the nurses for them to begin socialising as a nursing professional. Yet, it was indicated that student nurses should know how to behave and how to develop professionally without explicit teaching or information. It is through the use of nunchi as a social mechanism that the student participants could subjectively collect and interpreted information, including the discipline, norms and rituals of nurse groups, thus achieving professional socialisation. This is akin to the concept of intuitive reasoning. Although intuition is ‘a universal human experience’ [32], a study argued that while Westerners were more likely to use formal reasoning, East Asians were more oriented towards intuitive reasoning based on experience that has been contextualised, and not bound strictly by rules and logic [33].

The commonality between the above learning experiences in clinical contexts was notably not outlined in the formal curriculum for clinical education. Rather, the students learnt about the challenges and how to cope with them on their own by undertaking clinical placements. Dewey [34] claimed that collateral learning was often more crucial than the formal curricula, which indeed concurs with how the students in this research felt when reflecting on their clinical placement experience. The nurses and lecturers in this research also assume that nursing students would already know how to behave in clinical contexts, indicating that the nursing students’ professional socialisation process in the hierarchical clinical context was unplanned and unintended learning. In the literature, this unintentional and unplanned learning has been discussed under the concept of a hidden curriculum. Similar to this research, other studies [35–37] have reported that students in healthcare fields learn professional attitudes, values, norms and rituals through the hidden curriculum of their clinical education. The possibilities in achieving professional socialisation through the hidden curriculum indicates a need for nursing educators to reflect on our own nursing programmes. By addressing the hidden curriculum, nursing educators will also be able to learn how to best facilitate students’ professional development in clinical education, something that cannot be taught in a classroom or simulated learning environment [38, 39].

Despite the hidden curriculum’s significant influence on nursing students’ learning and professional socialisation during clinical education, there is still insufficient awareness of the curriculum and few attempts are made to include it in a formal curriculum [6]. To be able to carry out strategies aimed at addressing the hidden curriculum, it is imperative first to characterise it [36].

### Strengths and limitations

Explorations of the hidden curriculum in the nursing field are limited [35] despite its strong influence on clinical education. Moreover, studies that are concerned with it in healthcare fields rely solely on theoretical assumptions rather than empirical evidence. [35]. The present study was rigorously conducted using a qualitative research approach as discussed earlier in the rigour part and will contribute to the knowledge base of the hidden curriculum in clinical nursing education. The findings of this research will not be easily applicable to understand students’ professional socialisation processes in other socio-cultural contexts (e.g., Western countries). However, there is likely value in using the findings in other geographical regions that share similar cultural and contextual backgrounds to South Korea (e.g., China and Japan) (i.e., transferability).

## Conclusion

This research offers insight into nursing students’ experiences of their learning and professional socialisation during clinical placements and is based on empirical evidence. It also acknowledges the presence of the hidden curriculum through which nursing students achieve professional socialisation and obtains learning opportunities for skill acquisition.

Valuable lessons from this research can be used to inform the standard curriculum and assist with navigating the hidden curriculum. While experiential learning is a great opportunity for students to build on their coping skills and professional socialisation, this study identified that a lack of adequate support risks failure to manage both the formal and hidden curriculum, negatively impacting on students’ preparedness to cope with the demands of professional nursing role. Nursing educators therefore need to orientate students to the professional culture prior to beginning clinical placements. Characterising the hidden curriculum, and then incorporating it into a pre-placement programme can prove to be mentally and emotionally beneficial for students as they begin their clinical placements. This can be particularly useful for countries who share similar socio-cultural contexts as Korea.

More importantly, there needs first to be increased awareness and dialogue about the hidden curriculum in clinical nursing education, and its significance fort student learning. Further work can then begin on utilising the hidden curriculum to maximise students’ learning and prepare them for their professional careers.

## Additional file

Additional file 1: Interview guide. The interview guide that consists of semi-structured open-ended questions was used for the initial interview of the qualitative research to understand professional socialisation of nursing students in a collectivist culture. The development of interview guide was guided by the constructivist grounded theory. (DOCX 20 kb)

## Data Availability

Not applicable. The participants of this research did not consent to share their interview data to the public.

## Abbreviation

CGT: Constructivist grounded theory
