# Supplementary material for: Professional socialisation of nursing students in a collectivist culture: a qualitative study
Source: BMC Med Educ. 2019 Jul 9;19:254. doi: 10.1186/s12909-019-1690-z (PMC6617906; doi:10.1186/s12909-019-1690-z)
Supplement: Supplementary file 1 — Interview guide. The interview guide that consists of semi-structured open-ended questions was used for the initial interview of the qualitative research to understand professional socialisation of nursing students in a collectivist culture. The development of interview guide was guided by the constructivist grounded theory. (DOCX 20 kb) [file 12909_2019_1690_MOESM1_ESM.docx]

**Interview guide for nursing students**

**Initial open-ended questions**

1. What made you study nursing?
2. Can you describe the nursing education you have received (in South Korea)
3. Tell me about your experience of your nursing education

**Intermediate questions**

1. Can you describe the clinical contexts in which you have done placements?
2. Can you describe your roles during the placements?
3. As a student, what do you think you can and can’t do during the placements?
4. What do you think are the factors that have a positive or negative effect on your nursing education, especially clinical placements?
5. Who has the most influence on your learning in clinical placements? Why do you think so?
6. Tell me about your role model in nursing.
7. Tell me about your relationship with nurses and other medical personnel during your placements
8. What do you think about the nurses’ culture?
9. How do you feel when you go to clinical contexts for your placements, compared to when you were being taught in classrooms?
10. When you face any barriers during the placements (if any), how do you address it?
11. What do clinical placements mean to you?
12. What is your definition of nunchi?
13. Why do you need nunchi during your placement?
14. Tell me about nunchi in other occasions other than clinical environments

**Ending questions**

1. Is there anything else you think I should know to understand your clinical placement experience better?
2. Is there anything you would like to ask me?

**Interview guide for nurses**

**Initial open-ended questions**

1. Can you tell me about the clinical environments and the atmosphere of where you have worked?
2. How do you feel about working in those environments?
3. Tell me about your experience of supervising nursing students when they come for clinical placements.

**Intermediate Questions:**

1. What do you think the purpose of clinical placements is?
2. How do you teach the students when they come for their clinical placements?
3. What do you think are effective educational methods for the nursing students on their clinical placements?
4. What do you think about the students’ (social) position during their placement?
5. Tell me about nunchi and students’ use of the nunchi.
6. What kinds of clinical environments would be useful for nursing students to have effective placements?

**Ending Questions**

1. Is there anything else you think I should know to understand your experience of clinical placement supervision better?
2. Is there anything you would like to ask me?

**Interview guide for lecturers**

**Initial open-ended questions**

1. Tell me about the nursing education system in Korea, particularly clinical placements
2. In your opinion, what are the good and bad aspects of the current nursing education system?

**Intermediate Questions:**

1. Can you explain the current clinical placement curriculum? How is it run/conducted?
2. What do you think is the purpose of clinical placements?
3. What do you think about the clinical contexts that nursing students are currently doing their clinical placements in?
4. What do you expect nursing students to learn within the clinical context?
5. What do you think are the influencing factors that facilitate or hinder nursing students’ learning in clinical context?
6. What kinds of attitudes do you expect from nursing students in clinical contexts, and why do you think so?
7. What kinds of competencies do you expect nursing students to be equipped with to become a nurse? Why do you think so?
8. Tell me about nunchi and students’ use of the nunchi in clinical contexts.

**Ending Questions**

1. Is there anything else you think I should know to understand your nursing education experience and students’ experience of clinical placements better?
2. Is there anything you would like to ask me?
